# Finding cancer in mammograms: if you know it’s there, do you know where?

**DOI:** 10.1186/s41235-018-0096-5

**Published:** 2018-04-18

**Authors:** Ann J. Carrigan, Susan G. Wardle, Anina N. Rich

**Affiliations:** 1 Perception in Action Research Centre & Department of Cognitive Science, Macquarie University, Sydney, Australia; 2 ARC Centre of Excellence in Cognition & Its Disorders, Macquarie University, Sydney, Australia; 3 Centre for Elite Performance, Expertise, and Training, Macquarie University, Sydney, Australia

**Keywords:** Visual search, Medical imaging, Global processing, Breast density, Target detection, Target localisation

## Abstract

Humans can extract considerable information from scenes, even when these are presented extremely quickly. The ability of an experienced radiologist to rapidly detect an abnormality on a mammogram may build upon this general capacity. Although radiologists have been shown to be able to detect an abnormality ‘above chance’ at short durations, the extent to which abnormalities can be localised at brief presentations is less clear. Extending previous work, we presented radiologists with unilateral mammograms, 50% containing a mass, for 250 or 1000 ms. As the female breast varies with respect to the level of normal fibroglandular tissue, the images were categorised into high and low density (50% of each), resulting in difficult and easy searches, respectively. Participants were asked to decide whether there was an abnormality (detection) and then to locate the mass on a blank outline of the mammogram (localisation). We found both detection and localisation information for all conditions. Although there may be a dissociation between detection and localisation on a small proportion of trials, we find a number of factors that lead to the underestimation of localisation including stimulus variability, response imprecision and participant guesses. We emphasise the importance of taking these factors into account when interpreting results. The effect of density on detection and localisation highlights the importance of considering breast density in medical screening.

**Electronic supplementary material:**

The online version of this article (10.1186/s41235-018-0096-5) contains supplementary material, which is available to authorized users.

## Significance

In medical imaging, a radiologist searches and interprets a medical image to make critical diagnostic decisions (e.g. is that a cancer or not?), often under time pressure. With time and practice, experienced radiologists are thought to develop skills that allow them to form the basis of a diagnosis (normal or abnormal) during an initial glance at an image. This implies that the information extracted from the image in the first second of processing contains critical information that informs diagnosis. Here, we explore what type of information is present in this timeframe, particularly focusing on the presence (or lack thereof) of information about the location of potential abnormalities. We develop an image-level analysis of errors, which shows coarse location information exists in many apparently ‘incorrect’ location responses. Finally, we assess whether trials which imply detection of a target without localisation could be due to guessing. We demonstrate that for breast masses there is information that supports both detection and localisation of abnormalities, with better performance in images with low relative to high breast density. Our findings emphasise the need for breast density to be considered in screening reports and radiologist training. Notification for the patient and clinician about breast density and potential cancer risk may have a significant positive effect on outcomes, such as the provision of more suitable imaging modalities, and an earlier cancer diagnosis.

## Background

As soon as we open our eyes, our visual system processes an enormous amount of information in a short space of time. Early findings showed that an exposure of 100 ms is sufficient to extract the basic meaning of natural scenes (e.g. indoor vs outdoor; Potter, 1976). Using backward masking to precisely control for exposure times, others have shown that the distinction between natural scene categories at the superordinate level (e.g. manmade vs natural) and basic level (e.g. coast vs city) can occur with presentation durations as short as 20 ms (Greene & Oliva, 2009; Joubert, Rousselet, Fize, & Fabre-Thorpe, 2007). Furthermore, when primed with a category (e.g. animal or truck), objects can be detected at brief durations (Thorpe, Fize, & Marlot, 1996; VanRullen & Thorpe, 2001). This fast visual processing has also been reported among those who are experienced in domain-specific tasks such as medical imaging (Evans, Georgian-Smith, Tambouret, Birdwell, & Wolfe, 2013; Evans, Haygood, Cooper, Culpan, & Wolfe, 2016; Kundel & Nodine, 1975; Nodine et al., 1999). Kundel and Nodine (1975) showed that when presented with a chest radiograph for 200 ms, radiologists could detect an abnormality with 70% accuracy. Kundel, Nodine, Krupinski, and Mello-Thoms (2008) have since shown that within 1 s of viewing a mammogram, experts fixate on 67% of breast cancers (Kundel et al., 2008). Furthermore, when shown briefly presented mammographic displays (250 ms), radiologists can discriminate normal from abnormal at levels better than guessing (Evans et al., 2013, 2016). The evidence that observers can extract information with fast presentations from natural scenes (e.g. Potter, 1976; VanRullen & Thorpe, 2001) and medical images (e.g. Evans et al., 2013; Kundel & Nodine, 1975) suggests that the processing involved in early visual search is similar whether the display is a natural scene or a medical image, at least for experts.

Radiologists develop expertise in ‘visual search’ in such images over a period of years. It has been suggested that specialised training and ongoing experience leads to perceptual and cognitive ‘fine-tuning’ in the task of image interpretation (Nodine & Mello-Thoms, 2010). Maintaining such expertise requires interpreting high volumes of cases. For example, mammographic screening radiologists interpret more than 2000 cases per year (Rawashdeh et al., 2013). It is possible that expertise can be attributed to implicit learning and many hours of training and practice has allowed for the efficient guidance of attention to relevant regions in an image (Drew, Evans, Võ, Jacobson, & Wolfe, 2013). There is evidence that this extensive experience modulates the perceptual/cognitive system of experts: experienced radiologists outperform novices and trainee radiologists on tasks such as detecting an abnormality in brief images (Evans et al., 2013; Nodine et al., 1999), and in different patterns of eye movements between experts and novices. For example, Kundel and La Follette Jr (1972) compared the visual scan patterns of expert breast radiologists with trainees interpreting mammograms and found that the experts fixated on lesions faster and concluded search earlier than the novices. Others have shown that experts fixate true abnormalities within 1–2 s of image onset and most of their subsequent scanning is to confirm that there are no other lesions (Mello-Thoms et al., 2005). This follow-up takes about 5–10 s after initial fixation, after which a diagnostic decision is reached. There is an enormous amount of information that is processed in the first second of viewing a scene or image, so it is important that we understand the cognitive underpinnings of early visual search.

Kundel and Nodine (1975) developed a model that describes two distinct processes leading to a diagnostic decision. The first glance supports a global, or holistic, overview of the image, which indicates on a basic level whether the image deviates from a cognitive representation of a normal anatomical schema. The information extracted at this first stage is then proposed to constrain and guide search to the region of the image containing the abnormality (the second stage). For this to occur, the global signal must be informative about the location of the abnormality.

Recently, an alternative perspective has been offered by Evans et al. (2013, 2016). They suggest an initial abnormal signal could act to alert a radiologist that *something* is abnormal but without containing location information. Rather than guiding search to a location, this global signal then *changes the search strategy* to a more complete search for the abnormality. The initial signal could be supported by the rapid extraction of the summary statistics of the image, such as average orientation and size. In the basic vision literature, two stage models (e.g. Wolfe, Võ, Evans, & Greene, 2011) describe an initial, non-selective pathway which, although limited in capacity, extracts summary statistics in parallel from the display. In the model, global processing occurs along this pathway. A second, selective pathway recognises one or a few objects at a time and requires selective attention. Together these pathways combine to support perception. Evans et al. (2013, 2016) suggest that information via the non-selective pathway could alert a radiologist that something is abnormal, but the fine-grained detail, such as its location, only becomes available at the later selective stage.

Evans et al. (2013) compared the performance of radiologists and novices on the detection and localisation of abnormalities in mammograms. The stimuli were bilateral (left and right breast) mammograms where one side could contain subtle masses and architectural distortions that varied in size (10–48 mm). Such pathologies are highly variable and are difficult to detect and locate even by expert radiologists under free-viewing conditions. As a result, these have the highest reported rate of false negatives (Knutzen & Gisvold, 1993). Despite these difficult images, Evans et al. (2013) found that radiologists (but not novices) could detect an abnormality above chance (Mean *d′* was ~ 0.7 for 250 ms duration and up to ~ 1 for 2000 ms duration, where *d’* of 0 is chance). For the combined detection and localisation task, images were displayed for 500 ms. Following detection, the radiologists viewed a blank outline of the mammogram and were asked to localise by marking the abnormality with a mouse-click. Chance was determined by calculating the average percentage (across images) of overall tissue area lying within a predetermined region of abnormality. Although abnormalities could be detected by radiologists above chance at 500 ms, localisation performance was at chance. Evans et al. (2013) interpreted these results as evidence that the information extracted to support detection at brief durations does not contain location information but is rather based on an overall ‘gist’ or holistic signal. In a subsequent paper, Evans et al. (2016) did another series of experiments using mammograms, replicating and extending their initial findings. In their second experiment, they presented radiologists a set of 120 single-sided (one breast) mammograms for 500 ms and asked them to detect and then localise an abnormality. The unilateral mammograms either contained an abnormality (target-present), had no abnormality (target-absent), or was the contralateral breast from the target-present mammogram (no abnormality). In this experiment, mean *d′* for detection was 1.16 for the target-present/target-absent images, significantly above chance (0), whereas localisation accuracy was not significantly greater than that expected by chance (6%). They concluded that the radiologists could not localise a lesion despite detecting it. Further, they suggested that experienced radiologists could even make such judgements based on images from the contralateral (thus far normal) breast (remaining 40 images). Mean *d′* was 0.59 for detection of abnormality in the contralateral breast from a woman with signs of cancer in the other breast. This result is striking because the mammogram on which the judgement was based had no mass. These results provide intriguing hints that the information required for detection and that for localisation could be dissociable.

Evans et al. (2013, 2016) interpret their results as reflecting a global signal of abnormality that lacks information about the location of a specific mass. Indeed, the remarkable findings that a diagnosis could be made from the contralateral apparently normal breast when the opposite side was abnormal might be explained by this interpretation. There are, however, some alternative interpretations that need to be carefully considered and ruled out. First, to interpret a null effect as evidence for there being no effect (in this case no localisation), would need alternate statistics, such as a Bayes Factor (Dienes, 2011), to assess the degree of evidence for ‘no effect’. Second, the summary statistics (e.g. average *d* prime) could be inadequate to answer the key questions. For example, if participants click slightly outside the lesion, this would be categorised as incorrect, which would lead to the erroneous inference that there was no localisation information, whereas an analysis of the apparent error would clearly show localisation information. We also need to ensure that the abnormal images do not include ‘distracting’ features that could potentially be the basis of an apparently correct ‘abnormal’ response. Finally, in a detection experiment there will always be some ‘lucky guesses’ that are correct.

We need to consider the impact of these on the apparent dissociation between detection and localisation. The two studies by Evans et al. (2013, 2016) raise important questions, but the challenge to the Kundel and Nodine (1975) model of radiologists’ diagnostic decision-making rests heavily on the lack of information about the location of an abnormality. Here, we go beyond the summary statistics and explore image level variability, precision of localisation responses and the potential influence of guesses to test whether detection is possible without localisation.

The aims of the present study were to extend previous work by Evans et al. (2013, 2016) and explore in detail whether detection and localisation are dissociable. The claim that radiologists can detect the presence of an abnormality without knowing where it is has strong theoretical implications. Instead of the intuitive notion that the information in the first glance guides attention and the eyes towards the location of the potential abnormality, it implies a quite different process. Here, our first aim was to see whether expert readers of mammograms viewing brief displays can extract location information when a mass is either obvious or subtle. Female breast tissue is highly variable in mammographic breast density (MBD; Li et al., 2013), which provides us with a natural variant for manipulating the salience of a mass. In the human population, 40% of women aged 40–74 years have dense breasts (Sprague et al., 2014). Critically, as MBD increases there is a four- to sixfold increased risk of breast cancer (Boyd et al., 2010) and studies have shown that higher levels of MBD reduce radiologist sensitivity, thus limiting early detection of breast cancer (Al Mousa, Ryan, Mello-Thoms, & Brennan, 2014). For a radiologist, MBD increases the complexity of the image and could mask and/or distract from existing pathology. Our second aim was to explore the effect of breast density (which can make masses more difficult to see) on the type of information that can be extracted in a brief display. Finally, the distinction between theories rests heavily on the dissociation between detection and localisation of masses. Our third aim was therefore to develop methods that can test for evidence of this dissociation. To this end, we looked at the images in detail to explore the degree and source of localisation errors on apparent detection-correct trials, as well as considering the potential influence of ‘lucky’ guesses to ‘detection without localisation’ performance.

We investigate detection and localisation performance for a single mass in unilateral mammograms presented centrally for a brief duration and then masked. There is evidence of a bias to click directly in front of fixation (centre of the image) when the location is unknown (Buswell, 1935; Tatler, 2007). However, the mass location varied within the breast in our images, which minimises the influence of any such bias (i.e. a random central click is not likely to fall within the mass location). We presented two sets of mammograms that varied on density (high density and low density) and mass presence. As half of the images contained a mass that would be difficult to detect, we used two durations (unique images in each): 250 ms (within the timeframe others have considered to support gist-level information in medical images; Evans et al. (2013)) and 1000 ms (presumably well beyond gist level of perception). The participants performed a detection and an ‘exact click’ localisation task similar to Evans et al. (2013). We had two conditions for our target-present stimuli, each containing a single mass: a difficult condition (50%) in which the mass was subtle due to level of breast density and an easy condition (50%) in which the mass was obvious. The difficult condition is comparable to those of Evans et al. (2013, 2016). We predict that mass detection and localisation will be more accurate for mammograms with low density compared with those with high density at both experimental durations. We consider image variability, response imprecision and we use alternative analyses and a guessing correction to fully test for a dissociation between knowing an abnormality is present vs knowing where it is.

## Methods

### Participants

Twelve participants with experience in interpreting mammograms were recruited from BreastScreen New South Wales and local radiology practices (6 female, average age = 54 years, SD = 13 years). We defined experts as having at least four years of experience and in their current practice reading at least 2000 mammographic cases per year (Rawashdeh et al., 2013). The BreastScreen doctors (*n* = 11) read > 3000 mammographic cases per year, but we did also include one breast physician who reads > 1000 cases per year, as she had extensive experience (ten years). The average experience reading mammograms of our participants was 22 years (SD = 13 years). All gave informed consent and reported normal or corrected-to-normal vision. The study was approved by the Macquarie University Human Research Ethics Committee (Medical Sciences).

### Design, stimuli and apparatus

We used a Density (low, high) × Duration (250, 1000 ms) within-subjects design.

The stimuli were 160 full-field, de-identified, medio-lateral oblique digital breast mammograms obtained from the Dokuz Eylul Mammography Set (DEMS; Bulu, Alpkocak, & Balci, 2013), which varied on target presence/absence and high MBD/low MBD. Half the images (80) were normal and half contained a single mass previously diagnosed and coded according to the Breast Imaging and Reporting Data System (BIRADS; American College of Radiology: Breast Imaging Reporting and Data System Atlas. Reston, VA: © American College of Radiology, 2013). BIRADS is a standardised breast assessment tool developed for mammography that ranges from 0 to 6. In clinical practice, a radiologist assigns a BIRADS score to each image, which determines the next step in the diagnostic protocol. The 80 normal images had a previously assigned BIRADS code of 1 (no significant abnormality). The abnormal breast images consisted of BIRADS coded 2 (benign), 3 (probably benign), 4 (suspicious abnormality and biopsy recommended), 5 (highly suggestive of malignancy) and 6 (known pathological proven malignancy). The average size of the mass was 26.70 mm (SD = 13.23 mm) and the range was 8–54 mm. The mean distance from the centre of the screen to the mass border was 2.5° of visual angle (SD = 2°, range 0–8°). (Note: fixation was not controlled during the trial).

**Fig. 1:**
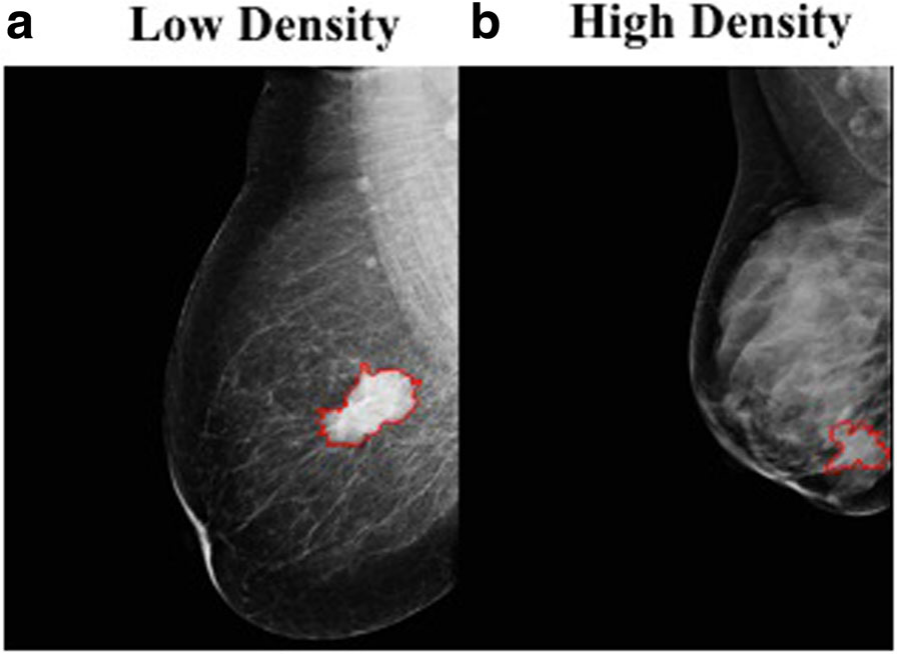
Exemplars of target-present images. The *red outline* depicts the mass (and did not appear in the actual stimuli). **a** Low-density breast that contains predominately fatty tissue, which is radio-translucent or black/grey. The higher contrast mass is easily seen. **b** High-density breast that contains normal fibroglandular tissue resulting in a more difficult search. The X-ray beam is attenuated by this tissue and appears radio-opaque or white on a mammogram

From this set, ten images were ‘cleaned’ using GraphicConverter (version 9.4). Image artefacts such as side markers and occasional dust speckles outside of the breast and large calcifications within the tissue were removed. One of the most challenging aspects of studying radiologists and using medical images rather than using artificial stimuli is that the human body varies widely anatomically. Stimuli were selected that contained only a single mass (so those with a second lesion were excluded). Difficulty was manipulated by including two sets of mammograms (dense: high MBD; fatty: low MBD) where half of the mass images (40) and half of the normal images (40) had high MBD. The remaining images had low MBD (see Fig. 1). Density was categorised on a dichotomous scale (low/high) by an experienced radiologist blind to the purpose of the study (M.B.) and one author with experience reading mammographic images (A.C.). These ratings were significantly correlated (*r* = 0.9, *p* < 0.0001).

The experiment was presented on a Macintosh MacBook Pro using MATLAB 2011B with the Psychophysics Toolbox Version 3 (Brainard, 1997; Pelli, 1997). The stimuli were centred on a 1920 × 1080 resolution 24-in., LG W2442PA, liquid-crystal display screen, refresh rate of 120 Hz. The participants sat approximately 70 cm away from the screen. The original resolution of the single mammograms was 4096 × 3328 or 3328 × 2560 pixels, which were downsized to 19° × 24° (18 out of 160) or 20° × 24° of visual angle. To validate our image categories and presentation durations, pilot data were collected from three radiologists at 250 ms and 500 ms durations two months before their participation in the experimental session. Previous studies which have used medical images have reported that a time-lapse of around two months between each session reduces the likelihood of recall (Berbaum et al., 2015). On the basis of these pilot data, we increased the long duration condition to 1000 ms.

### Procedure

**Fig. 2:**
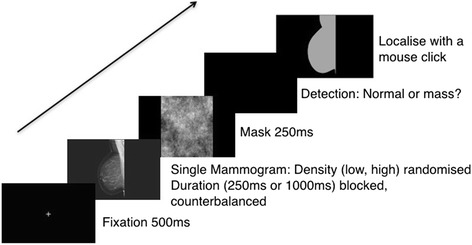
Example trial for 12 radiologists who were asked first whether the image was normal or contained a mass, and then to use the mouse to indicate the location of the mass if present

The experiment was conducted onsite at various metropolitan Sydney BreastScreen and radiology practice locations. We presented the stimuli at two presentation durations (250 ms, 1000 ms) in separate blocks, counterbalanced in order across participants. For each participant, the particular image presented in each duration was randomly selected without replacement. After four practice trials at 2000 ms with feedback and a further six trials at the experimental durations (three at 250 ms, three at 1000 ms; blocked) with feedback, the radiologists viewed 160 trials without feedback. The radiologists were asked to detect ‘any mass that you would recommend for further investigation’. Each trial began with a fixation point for 500 ms, followed by a centrally presented left medio-lateral oblique breast image. This was followed by a backward 1/f noise mask for 250 ms after each stimulus presentation and a black screen asking the radiologists to categorise the mammogram using a key press as either ‘normal’ (left arrow key) or ‘mass’ (right arrow key), followed by a black screen with a grey mask of the breast (each unique mammogram was paired with its corresponding mask). The radiologists were asked to ‘please click with the mouse the exact location where you saw a mass’. In the case of normal responses, they were asked to click anywhere on the display. There were 20 trials per condition (duration/target presence/density). Figure 2 shows the trial sequence. Participants began the next trial with a key press.

### Analysis

Following the recommendations of Cumming (2012), we present Mean differences (M_diff_) with 95% confidence intervals (CI), as well as a Cohen’s *d* estimate of effect size corrected for small sample size, to assist in accurate interpretation of the effects. This latter measure, *d*_*unb*_, represents an adjusted, unbiased Cohen’s *d* standardised effect size applied to single sample t-tests where *d*_*unb*_ = (1 - 3 / (4*df - 1)) * *d* (Cumming, 2012).

## Results

The aims of the experiment were to see whether expert readers of mammograms viewing brief displays: (1) can extract location information; (2) are affected by breast density in the type of information that can be extracted; and (3) show a dissociation between detection and localisation.

### Detection accuracy

**Fig. 3:**
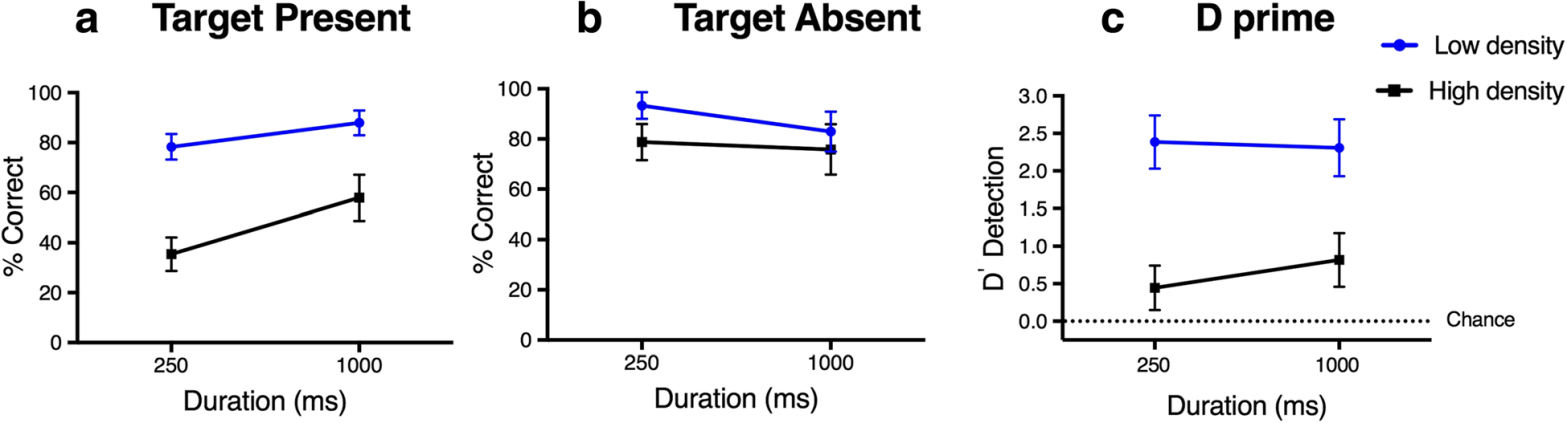
Detection performance. **a** Average percentage correct on target present trials. **b** Average percentage correct on target absent trials. **c** Average d′ on the detection task. *Error bars* represent 95% confidence intervals

First, we calculated accuracy for target present and target absent trials to test whether the radiologists could detect a mass at these durations. Figure 3 shows performance on the detection task presented as accuracy for target present and absent trials separately (Fig. 3a and b) and sensitivity (Fig. 3c). Figure 3a shows better performance for the low-density images (more obvious masses) than the high-density images (where the masses are more difficult to find even in free-viewing). Accuracy also improves with duration. Figure 3b shows accuracy for the target absent trials. The radiologists appeared less accurate on target absent trials at the longer duration, showing they tended to make false alarms when given slightly more time to inspect the display.

D prime was calculated as a function of abnormality present or absent. Higher *d′* indicates greater sensitivity: the higher the *d′*, the more accurately the radiologists responded to both target present and target absent trials (i.e. reported a mass when a mass was present *and* no mass when no mass was present). A *d′* of zero indicates there is no sensitivity and the participant is performing at chance (i.e. no better than guessing).

Figure 3c presents the *d′* data. Single sample t-tests (Bonferonni adjusted, alpha = 0.0125) on average *d′* relative to 0 (chance) for each duration and density showed that radiologists do have information about the presence of the mass at both durations. Performance at 250 ms for the low-density condition was greater than chance (*t*(11) = 14.97, *p* < 0.0001, M_diff_ = 2.39, 95% CI = 2.03–2.74, *d*_*unb*_ = 5.69) as was performance in the more difficult high-density images (*t*(11) = 3.3, *p* < 0.007, M_diff_ = 0.44, 95% CI = 0.15–0.74, *d*_*unb*_ = 1.3). As one might expect, this was also the case at the longer duration of 1000 ms, both for low-density images (*t*(11) = 13.38, *p* < 0.0001, M_diff_ = 2.31, 95% CI = 1.93–2.69, *d*_*unb*_ = 5.09) and high-density images (*t*(11) = 5.04, *p* < 0.0001, M_diff_ = .82, 95% CI = 0.46–1.17, *d*_*unb*_ = 1.92). Although high-density *d′* values reflect poorer performance than seen in free-viewing, where radiologists have *d′* values around 2.5–3.0 (D’Orsi et al., 2013), performance already approaches these levels for the low-density images, even at 250 ms (see Fig. 3c). These results suggest that when the mass is relatively easy to see (low density), *d′* in the first quarter of a second is already close to that of free-viewing.

As one would expect, we can see from Fig. 3c that performance for the low-density images is better than the high-density images. This obvious pattern was confirmed by a repeated measures ANOVA with the factors of Density (low, high) × Duration (250, 1000) on the mean *d′* values. This showed a main effect of Density (*F*(1, 11) = 133.51, *p* < 0.0001, *η*^2^_*p*_ = 0.92), no effect of Duration, (*F*(1, 11) = 0.98, *p* = 0.344) and no Density × Duration interaction (*F*(1, 11) = 2.09, *p* = 0.18).^1^

### Localisation accuracy

**Fig. 4:**
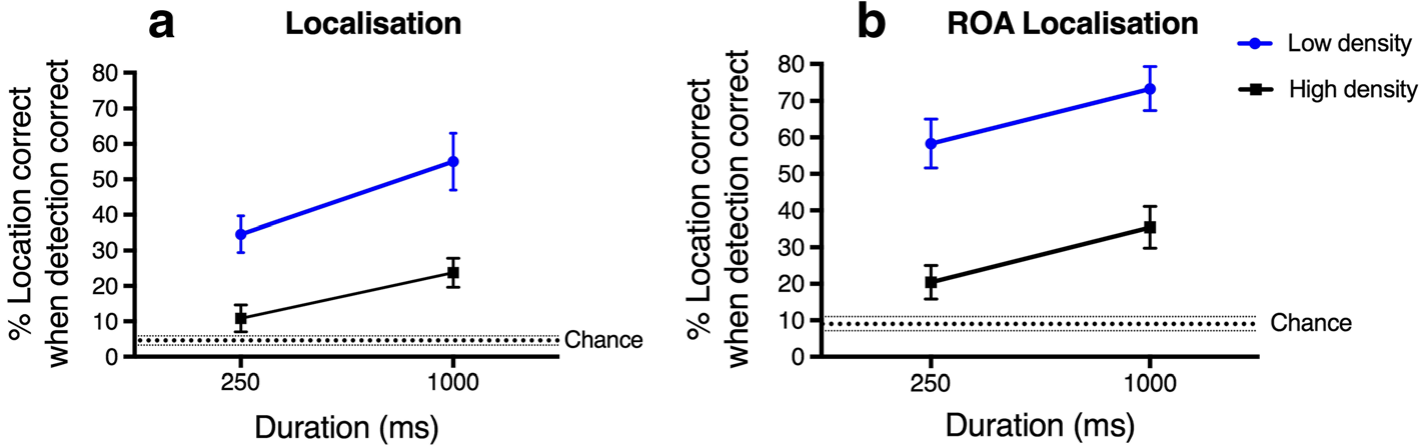
Detection and localisation results. **a** Average percentage correct on the localisation task for trials when detection was correct; **b** Average percentage correct on the localisation task when a region of acceptance (ROA) around the lesion is included. Chance is 4.4% and adjusted to 9.1% when including the ROA (*dotted line*) with 95% confidence intervals. *Error bars* represent 95% confidence intervals

Our key questions were: first, whether there is localisation information when detection is correct; and, second, how breast density influences localisation. Using the same method as Evans et al. (2013, 2016, Experiment 2), we compared the location of the mouse click with the location of the actual mass and coded the response as either accurate (participant clicked on or within the boundaries of the mass) or not (any other location). We analysed trials where the participants were correct on detecting an abnormality at each exposure duration (i.e. correct detection target-present trials). We compared localisation performance to chance, calculated across the 80 target-present images as 4.4% (95% CI = 3.02–5.75). This is the proportion of breast tissue that contains the mass relative to the proportion of total tissue; thus, it represents the average number of possible random locations radiologists could select, taking into account the lesion and image size across all of the target-present images. Figure 4a shows the percentage of trials when the radiologists responded correctly on localisation task, when detection was correct, for low density (blue line) and high density (black line) at the two durations, compared with chance. Single sample t-tests (Bonferroni adjusted, alpha = 0.0125) showed that radiologists’ localisation accuracy was significantly above chance (4.4%) for 250 ms presentations of low-density images (*t*(11) = 12.9, *p* < 0.0001, M_diff_ = 30.18, 95% CI = 25.03–35.33, *d*_*unb*_ = 4.9) as well as for high-density images (*t*(11) = 3.74, *p* = 0.003, M_diff_ = 6.43, 95% CI = 2.64–10.22, *d*_*unb*_ = 1.42). The same pattern was evident at the longer duration of 1000 ms for low (*t*(11) = 13.9, *p* < 0.0001, M_diff_ = 50.6, 95% CI = 42.59–58.61, *d*_*unb*_ = 5.28) and high (*t*(11) = 10.41, *p* < 0.0001, M_diff_ = 19.35, 95% CI = 15.26–23.44, *d*_*unb*_ = 3.95) density images.

To investigate the effect of density on localisation (Fig. 4a), we conducted a repeated measures ANOVA with the factors of Density (low, high) × Duration (250, 1000) on the mean percentage localisation correct values from the correct detection target-present trials. Again in line with expectations, this showed a main effect of Density, with better localisation accuracy in the low- than high-density conditions (*F*(1, 11) = 114.07, *p* < 0.0001, *η*^2^_*p*_ = 0.91), a main effect for Duration, with better localisation accuracy at 1000 ms than 250 ms (*F*(1,11) = 53.01, *p* < 0.0001, *η*^2^_*p*_ = 0.83), and no Density × Duration interaction (*F*(1,11) = 2.17, *p* = 0.17). These analyses show that radiologists were statistically above chance in localising the target on trials where they successfully detected a mass. However, as localisation performance is far from perfect, we have some trials on which detection apparently occurred without localisation information being available. This could reflect a global signal as suggested in the previous literature (Evans et al., 2013) and to investigate this possibility thoroughly, we conducted several follow-up analyses.

Before concluding one has evidence of ‘detection without localisation’, there are some important alternatives to be considered. First, we would like to note that before concluding anything from a null localisation effect, we need to use statistics that can assess the evidence for *no effect* (no localisation when there is detection) rather than just no evidence. Frequentist statistics do not allow for the interpretation of null effects – a *p* value greater than alpha merely informs us that we do not have evidence to reject the null hypothesis. To see whether there is evidence *for* the null hypothesis of *no localisation* information, we could instead calculate a Bayes Factor (BF). In line with Jeffreys (1961), a BF < 0.3 indicates that the data support the null rather than the alternative hypothesis, a BF ~ 1 indicates maximal insensitivity of the experimental evidence, whereas a BF > 1 indicates the data support the alternative hypothesis (BF > 3 suggests evidence for the alternative) (Dienes, 2011). In our case, we do not have a null effect in any condition, but we can still calculate a Bayes equivalent of a single sample t-test compared to chance (4.4%) to illustrate the point: if we test just the difficult images that are comparable to those of Evans et al. (2013, 2016), we can see strong evidence for the alternative hypothesis that localisation information exists: for the high-density condition at 250 ms, the BF(12) = 14.73 and at the longer duration, 1000 ms, BF(12) = 31,052.09. Consistent with our frequentist statistics results, we conclude that the radiologists are localising targets better than chance in the high dense conditions.

**Fig. 5:**
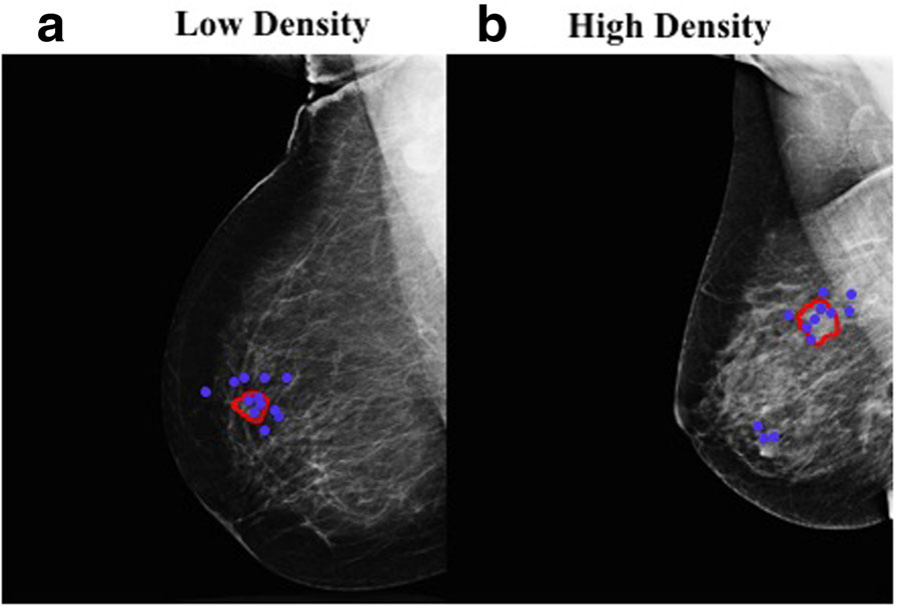
Exemplars from the target present stimuli set illustrating the mass (*red outline*, not shown in the experiment) and localisation responses of the 12 radiologists (*blue*) collapsed across duration. **a** Low-density image showing precision errors. The blue mouse-clicks for localisation show that the eight radiologists who were ‘incorrect’ on this image may have information about the location of the target. **b** High-density image showing the effect of a naturally occurring distractor. Three radiologists localised the distractor as the abnormality (note a further four ‘incorrect’ responses are near the mass (*red outline*) but imprecise)

Our second consideration is whether summary level statistics such as overall accuracy or sensitivity are adequate to address the ‘detection without localisation’ question. In fact, one cannot be sure of ‘detection without localisation’ without examining the error trials carefully. A null localisation effect could, for example, be due to less precision in the localisation task than the detection task due to the additional requirements rather than a true lack of localisation information. This could include decay in the visual short-term memory trace over time or motor error in clicking the precise location. If such factors influence the precision of the localisation responses, we should see localisation errors that nonetheless cluster around the correct region. Our radiologists were scored correct on localisation if the mouse-click occurred within or on the boundaries of the lesion, consistent with Evans et al. (2016) (Evans, personal communication, 2017). However, when we look at the incorrect localisation responses, we see that this does not accurately reflect the degree of localisation information. For example, in Fig. 5a, many of the ‘incorrect’ responses suggest the participant had some information about location.

There is also inherent variability in real-world stimuli. Although we carefully selected images with only one true mass and removed obvious image artefacts (e.g. dust), the images have naturally occurring variations in breast tissue. We need to examine the responses at an image level to assess whether such variance may have contributed to trials of apparent successful detection without accurate localisation. Figure 5b shows clearly an image where natural variability has contributed to three incorrect responses to a distractor in the breast (presumably in these cases, the radiologists were responding ‘abnormality present’ to this distractor, rather than the actual mass). The responses on these images suggest that apparent ‘detection without localisation’ can reflect coarse or less precise localisation, rather than no localisation, warranting image-level investigation.

To quantify the degree to which such examples might influence our results, we conducted a post-hoc image analysis collapsed across participants for each duration. We calculated the distance between the response click and the mass (i.e. the degree of incorrect localisation). In academic radiology, a region of acceptance (ROA) for lesion localisation is determined by taking into account the size of the largest lesion (e.g. Haygood et al., 2014). Following this convention, we measured the radius of the largest mass in the image set (27 mm) and added this value to the boundary values for all the target present images. Using this method, localisation is scored correct when a radiologist clicks within this ROA, allowing for a margin of response imprecision and reducing the ‘tightness’ of acceptance. We further examined the trials that were still incorrect to quantify the distance from the lesion boundary.

**Fig. 6:**
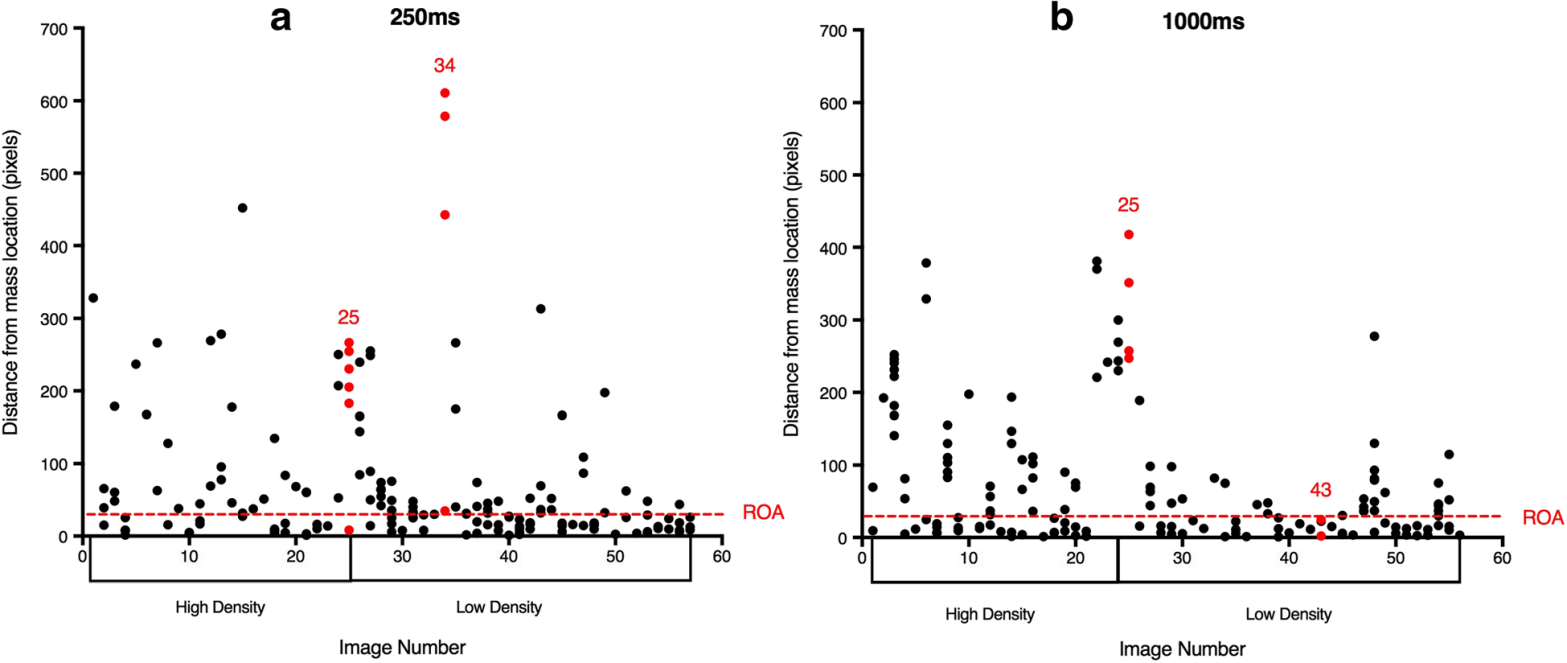
Localisation errors showing the distance between the localisation response and the mass for each image (detection correct target-present trials only). **a** 250 ms duration; **b** 1000 ms duration. The *x-axis* represents the images (divided by high and low density. Note: the image numbers are arbitrary for the purpose of the graph only). A correct score on localisation would score 0 (excluded from the figure). The *y-axis* is the distance (in pixels) from the mass border. The *dashed red line* represents the region of acceptance (ROA). *Red numbers* are data points in response to images with unusual characteristics: 25 (250 ms) is the high-density image presented in Fig. 5b showing the mouse-clicks on a distractor. 34 is a low-density image which contained a prominent lymph node in the axillary tail of the breast which appears to have captured four radiologists’ attention; 25 (1000 ms) is a low-density image containing a small mass and 43 is the low-density image presented in Fig. 5a showing the cluster of mouse-clicks near the correct location

Figure 6 shows image level analysis for the localisation data on incorrect trials plotted as a function of distance (in pixels) from the closest boundary of the mass, collapsed across radiologists (Fig. 6a: 250 ms; Fig. 6b: 1000 ms). Trials on which the detection response was incorrect are not included (250 ms: high density n = 12, low density n = 1; 1000 ms: high density n = 8; low density n = 0). Correct responses for localisation (when detection correct) would appear on the baseline and are also not included in the figure (250 ms: high density n = 3, low density n = 8; 1000 ms: high density n = 8; low density n = 8). The dashed red line represents the ROA plotted at 29 pixels. Figure 6 shows a considerable proportion of the clicks lie within this decision boundary and highlights how the variability within each image affected accuracy due to factors such as mass size and distractors.

### Localisation accuracy including a ROA

We calculated percent correct for localisation trials with an ROA included in assessing localisation for target-present trials with correct detection responses. Figure 4b shows the percentage of trials in which ROA localisation was correct for low-density (blue line) and high-density (black line) images across both durations, compared with chance. ROA chance was calculated as 9.1%, adjusted to account for the increased proportion of tissue included in the ROA. The summary-level measures clearly indicate better accuracy for all conditions compared with the non-ROA data (Fig. 4a), especially for the 250 ms high-density condition (ROA Mean = 20.42%; non-ROA Mean = 10.83%), suggesting that the Evans et al. (2013, 2016) method for calculating localisation may not adequately capture the degree to which location information is present.

This post-hoc analysis highlights the variability and challenges which exist when using real-world stimuli and the importance of carefully examining the data from individual images rather than stopping at summary statistics. These findings suggest that the apparent lack of localisation on some trials where a mass was detected is, at least in part, driven by image variability, such as small masses in a proportionally large breast and normal tissue with salient features (distractors), and response imprecision. When we apply a more liberal localisation ROA, we see evidence that coarse localisation information exists, with a higher proportion of correct localisation responses even for the more difficult images.

**Fig. 7:**
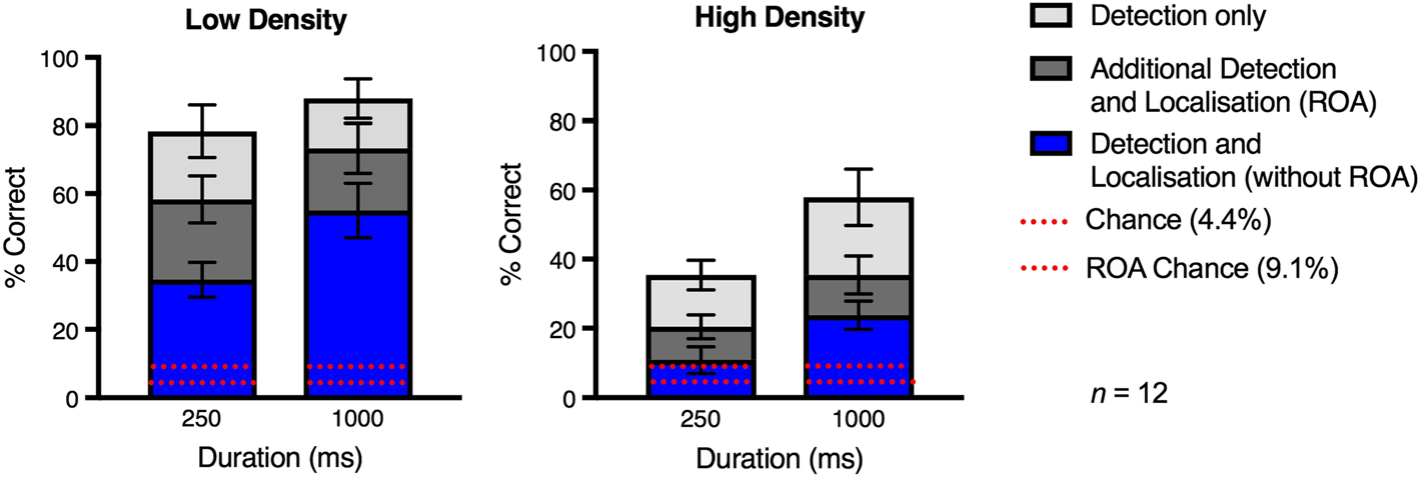
Percentage correct detection and localisation on target-present trials for low- and high-density mammograms plotted by duration (250 ms, 1000 ms). Data are separated by response accuracy: detection and localisation correct (*blue bar*); the additional proportion of trials where localisation is correct when a ROA is included (*dark grey bar*); and detection correct/localisation incorrect (*light grey bar*). *Error bars* represent 95% confidence intervals

We can also bin trials on which detection was correct according to their response profile to further examine the distribution of trial performance. Figure 7 shows the localisation data calculated using an ROA as a function of detection performance (collapsed across radiologists and images) for trials on which detection plus localisation were correct (blue bar), the additional localisation correct trials produced by including a ROA (dark grey bar) and detection only trials on which localisation was incorrect (light grey bar).

In addition to the trials with evidence for coarse localisation or precise mis-localisation, Fig. 6 shows some remaining trials on which localisation is clearly incorrect; these contribute to the light grey bars in Fig. 7. These trials could be evidence for ‘detection without localisation’, which seems key to interpretations of radiologists using ‘gist’ or a global signal. However, there is one final consideration before making such an interpretation: we need to be sure that the number of trials on which this occurs exceeds the rate at which such trials would occur simply from ‘lucky’ guesses. With any visual detection task, some proportion of trials will be correct by chance. A *d′* above chance shows more trials are correct than would be predicted by simply guessing, but if one wants to infer that there are trials in which there is ‘detection without localisation’, we need to calculate what proportion of these could be lucky correct detection guesses, followed by a localisation guess (which has less chance of being correct, recall chance was 4.4% for the non-ROA analyses and 9.1% for those including a ROA).

We calculated a guessing probability using the method described in Howe and Webb (2014). They were interested in whether observers could ever ‘sense’ a change in a change blindness paradigm without knowing where the change was. In their method, one works out what proportion of correct detection trials (in their study, detection of a change) could be due to lucky guesses by creating a hypothetical observer who can only detect a change when it also knows what that change is (i.e. there is no true detection without localisation, therefore any such trials are due to correct guesses). Here, we used the same logic, a hypothetical observer who cannot detect a mass without also knowing where that mass is, to work out the proportion of trials on which correct detection combined with incorrect localisation could be due to lucky guesses. We can then compare actual performance with this prediction for each radiologist.

$$ Calculated\;N\left( hypothetical\kern0.17em observer\right)=Q\left(Y- PA\right)/\left(1-P\right) $$

where  *Q *= proportion of possible incorrect localisations; *Y* = number of target present trials on which the participant responded ‘target present’ (hits); *P* = proportion of target absent trials on which the participant responded ‘target present’ (false alarms) and *A* = actual number of target present trials (note, there is no correction applied to an observer with no false alarms).

**Fig. 8:**
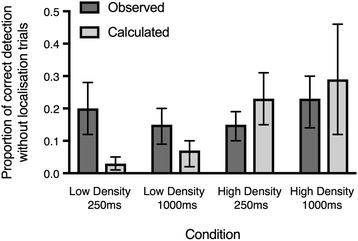
The proportion of correct ‘detection without localisation’ trials (*dark grey bars*) compared to the proportion of calculated (guessing) trials for a hypothetical ideal observer (*light grey bars*) for low- and high-density mammograms plotted by duration (250 ms, 1000 ms). *Error bars* represent 95% confidence intervals

We calculate a guessing probability for the ROA localisation data, as this already takes into account any slight imprecisions in the localisation responses, giving the most accurate view of localisation information at a summary level. If the actual participants correctly indicated the presence of a mass in the absence of a correct location response more often than this hypothetical observer, this provides evidence for information about the presence of an abnormality without knowing where it is: ‘detection without localisation’. Figure 8 shows the number of ‘detection without localisation’ trials from our data (dark grey bars) and the number of trials the hypothetical observer would ‘guess’ for all four conditions (light grey bars).

From Fig. 8, it is clear that there are only a small number of trials representing apparent ‘detection without localisation’, which makes statistical analysis unlikely to be reliable. However, even just from the graph one can see that only for the low-density conditions is there any chance that there might be more detection without localisation trials than predicted by our hypothetical observer. From the image level analysis, these trials could reflect errors accounted for by response imprecision (e.g. large amount of breast tissue/small mass) and distractors. Recall that it is our high-density condition that has images in which the mass is comparable in difficulty to Evans et al. (2013, 2016), making this the key condition. We have no evidence that for this high-density condition the number of observed ‘detection without localisation’ trials is more than what would be predicted by ‘lucky’ guesses.

## Discussion

The aim of this study was to examine the type of information that is available in the initial processing of a medical image (mammogram) by experienced radiologists, focusing on *detection* and *localisation* of potential abnormalities. We found radiologists were able to *detect* abnormalities at both durations (250 ms, 1000 ms) and density conditions (low, high), with a significant effect of duration. Overall summary statistics also supported the presence of *localisation* information, with the radiologists performing better than chance for both the 250 ms and 1000 ms durations, for the low- and high-density mammograms. Breast density affected performance in a predictable way, with better performance for low- than high-density images. As our key question related to a potential dissociation between detection and localisation, we carefully examined trials on which there seemed to be a dissociation. We suggest a number of factors that can lead to an underestimation of localisation information such as image variability, the precision of localisation responses and correct detection guesses. Overall, our data suggest that although it is possible that there may be a dissociation between detection and localisation on a small number of trials, particularly on easy trials (low density), there are other plausible explanations for the majority of such apparent dissociation trials.

Recent high-profile papers have concluded that radiologists can detect but not localise abnormalities in briefly presented mammograms (Evans et al., 2013, 2016). These papers suggest a different process to the previous theory that the information in the first glance guides experienced radiologists’ attention and directs their eyes towards the location of the potential abnormality (Kundel & Nodine, 1975). Specifically, Evans et al. (2013, 2016) proposed that the information extracted in the early signal is a global impression, which alerts the radiologist to the presence of an abnormality and then prompts a more thorough search, rather than guiding attention to the region of the abnormality directly. This alternative theory depends crucially on radiologists being able to detect masses in the *absence* of any information about location.

One of the key distinctions between the Evans et al. (2013, 2016) studies and our study is the type of abnormalities included. They presented ‘subtle masses and architectural distortions’ (Evans et al., 2013, p. 1172). This suggests there were a mix of potentially localisable abnormalities (subtle masses) and abnormalities with less well-defined locations (architectural distortions, which do not contain a discrete mass in the parenchyma). In our stimuli set, we only included images with a single localisable mass, which may have increased the likelihood of finding localisation information. Perhaps a global or gist signal supports detection separate from location when there is weak (or no) location information in the stimulus itself. This seems a plausible explanation for related intriguing findings in which radiologists are above chance in detecting an abnormality in a patient when shown whole mammograms of a contralateral normal breast (Experiment 2) or only a patch of a mammographic image that does not actually contain the mass (Evans et al., 2016, Experiment 4). In these cases, there is no ‘mass’ to localise, making these findings less relevant to the question of whether a localisable mass can indeed be detected without being localised (although obviously pertinent to the idea that a global signal can be used to diagnose an abnormality). It would be interesting to compare the localisation performance across different types of breast pathology to see whether there are sub-types of cancer for which experts are able to detect abnormalities based on gist without any location information (either because the type of abnormality has diffuse boundaries or because there is sufficient signal of abnormality in the overall image).

Even when we used a conservative measure of localisation (click within the mass boundary), we did not replicate the findings of Evans et al. (2013, 2016) that there are circumstances where radiologists can detect a mass above chance but not localise it. This could simply reflect that we were not at exactly the right durations to catch a dissociation due to variability in the experience of the participants, difficulty of the images and other cross-experiment differences between our study and those of Evans et al. (2013, 2016). Another potential factor that could influence the difference between the studies is that our participants were more experienced than those of Evans et al. (2013, 2016). This may be a reason that we found localisation at a summary statistics level: our more experienced participants could extract information more rapidly and therefore processed the images in greater detail. To make the inference that there is *no localisation*, however, still requires a number of additional steps, including using an approach such as Bayes statistics, rather than standard frequentist statistics. Here, we have outlined the steps that seem crucial to be able to make an inference of dissociation between detection and localisation.

Although at the summary statistic level we did not replicate the lack of localisation information, we did find trials on which detection responses were correct but those for localisation were incorrect. We were therefore able to use these to investigate factors that might contribute to an apparent dissociation between detection and localisation. First, variability in the target-present images might be contributing misleading data to the summary statistics. Using real-world stimuli rather than typical laboratory visual search displays allows for high ecological validity, but the available images tend to be highly variable and it is difficult to control for factors such as co-existing variables (e.g. breast calcifications, target number and size, and breast tissue type). Indeed, we identified images where there were clear clusters of incorrect localisation corresponding to a specific visual feature in the image (Fig. 5), suggesting the detection response was based on an incorrect identification (i.e. of the distracting feature). Second, we find evidence that coarse localisation information is often present in apparently incorrect responses. When we use a region of acceptance around the lesion, we see clusters of correct localisation responses surrounding the lesion. This suggests that task demands, such as having to hold the information through a detection response and subsequent location screen, may result in a loss of precision. Alternatively, it may be that the location information is only present at a coarse level in the first place (and is perfectly maintained). Finally, on trials where there is detection but incorrect localisation (by whatever definition one uses), it is important to consider the contribution of correct detection guesses. We used a method for estimating the effect correct guesses might have on the subsequent results. The key high-density condition, which is most similar to that of Evans et al. (2013, 2016), gives no evidence for there being more ‘detection without localisation’ trials than would be predicted to be lucky guesses. Thus, the pattern taken from a small number of trials suggest that in the difficult images, such as our set of high-density mammograms, apparent ‘detection without localisation’ responses can be accounted for by ‘lucky’ guesses.

Our only evidence of an apparent dissociation between detection and localisation comes from the low-density conditions. Intuitively, a salient mass seems most likely to have localisation information recorded, as there is a stronger bottom-up signal (much like a classic ‘feature search’). Indeed, we do see overall better performance in the low-density conditions compared with the high-density conditions (although nowhere near ‘pop-out’ performance). Although our ROA takes into account coarse localisation information, it cannot account for image-level variability where a distractor may have been selected or the potential decay of localisation information over time. Thus, while it is possible that these potential ‘detection without localisation’ trials in the low-density condition could reflect a global signal that is used to make a detection response, as proposed by Evans et al. (2013, 2016), these trials could alternatively reflect the contribution of other factors to reducing localisation accuracy. Overall, such ‘detection without localisation’ occurred on a very small number of trials (~ 4), precluding statistical analysis, which means we have only the numerical difference to support any such inference. This means that for most of our stimuli, including those most similar to the previous studies, when the radiologists reported detecting a mass, they also had some information about where it was.

The proposal by Evans et al. (2013, 2016) that radiologists use a global signal lacking in location information has important theoretical implications, as it identifies a very different mechanism from the Kundel and Nodine (1975) classic theory. Our results, however, demonstrate that successful detection of a mass in briefly presented mammograms is typically accompanied by information about location. This is more consistent with the Kundel and Nodine (1975) model: that the initial signal guides attention and eye movements to the lesion. To fully reconcile these distinctions, we need a study which investigates the presence (or lack thereof) of both global and localisable signals across three clearly defined conditions with different degrees of potential localisation (a salient mass, a subtle mass or diffuse parenchymal change). We then need to ensure that the analyses are appropriate to the key question of whether any localisation information exists through a thorough image-level analysis.

Both detection and localisation performance decreased with increased breast density at fast presentations. These results are related to what we know about clutter in natural scenes and visual search in free-viewing: increasing clutter or set size decreases performance (Adamo, Cain, & Mitroff, 2015; Asher, Tolhurst, Troscianko, & Gilchrist, 2013; Rosenholtz, Li, Mansfield, & Jin, 2005; Rosenholtz, Li, & Nakano, 2007; Whitney & Levi, 2011; Wolfe, 1994). Fibroglandular tissue, which increases density on a mammogram, appears more radio-opaque than fat and may increase crowding and/or masking effects reducing performance in the denser mammograms. In the medical perception literature, there have been a number of studies that have investigated factors such as lesion subtlety, which may be dependent on the surrounding anatomical structures (e.g. Krupinski, 2005). Analogous to clutter interfering with performance in natural scenes, our results show similar effects in radiologists interpreting medical images.

These findings improve our understanding of how density can influence a radiologists’ diagnostic decision and therefore have clinical relevance. Female breast tissue is highly variable with regards to MBD (Li et al., 2013) and high levels of breast density reduce radiologist sensitivity (see Al Mousa et al., 2014). It has been suggested that what radiologists perceive and thus report in the first second is critical (Mello-Thoms, 2009), that women with dense breasts make up almost a half of the population (Sprague et al., 2014), and that there is an increased risk of developing cancer in dense breasts (Boyd et al., 2010). Our results confirm that MBD has a negative impact on mass detection and localisation when radiologists are shown an image briefly. From a clinical viewpoint, we should inform women and their clinicians about their MBD levels, for appropriate and personalised care. For instance, in the case of a dense breast, further imaging modalities such as three-dimensional mammography (digital breast tomosynthesis), ultrasound or magnetic resonance imaging will help facilitate a definitive diagnosis. Although for almost half of the United States, density scoring is included (Slanetz, Freer, & Birdwell, 2015), current breast screening reporting protocols in Australia do not include a mammographic density rating. Our data show that high breast density reduces the amount of information available in the first glance, suggesting reporting this information should be mandatory.

## Conclusions

Here, we explored the degree to which information available in very brief presentations of medical images can support both detection and localisation of a mass in mammograms. Access to location information is crucial for guiding actions or further analysis (e.g. eye movements). We find a tight link between information supporting detection and localisation, using methods that allow a stronger test of the claim that detection of a mass can occur based on gist without knowledge of location. Although it is certainly possible that gist and the non-selective pathway of visual processing contribute to the detection of a non-localisable abnormality, our systematic examination of the factors that can result in apparent dissociation between detection and localisation demonstrates the importance of going beyond summary statistics when seeking to test this hypothesis. We emphasise the importance of considering factors such as stimulus variability, response imprecision and participant guessing. Our results are consistent with Kundel and Nodine’s (1975) model of radiologist visual search suggesting that the initial signal in a brief glance contains information that subsequently guides attention to the abnormality. Finally, we suggest the finding of reduced performance for dense mammograms illustrates the importance of reporting density information in the context of medical screening.

## Additional file

Additional file 1: **Figure S1.** Detection accuracy: percentage correct for individual radiologists on target present trials for (a) low-density and (b) high-density mammograms on the detection task. The three radiologists that had piloted the experiment previously are illustrated in red. **Figure S2.** Detection accuracy: percentage correct for individual radiologists on target absent trials for (a) low-density and (b) high-density mammograms on the detection task. The three radiologists that had piloted the experiment previously are illustrated in red. **Figure S3.** Detection accuracy: sensitivity (d′) for individual radiologists for (a) low-density and (b) high-density mammograms. The three radiologists that had piloted the experiment previously are illustrated in red. **Figure S4.** Detection and localisation results: percentage correct on the localisation task for individual radiologists on trials when detection was correct for (a) low-density and (b) high-density mammograms. The three radiologists that had piloted the experiment previously are illustrated in red. Chance is 4.4% and adjusted to 9.1% when including the ROA (dotted line) with 95% confidence intervals. **Figure S5.** Detection and localisation results: percentage correct on the localisation task when a region of acceptance (ROA) around the lesion is included for individual radiologists for (a) low-density and (b) high-density mammograms. The three radiologists that had piloted the experiment previously are illustrated in red. Chance is 4.4% and adjusted to 9.1% when including the ROA (dotted line) with 95% confidence intervals. (DOCX 1002 kb)

## Abbreviations

ANOVA: Analysis of variance
MBD: Mammographic breast density
SD: Standard deviation

## Footnotes

1 Individual observer data can be found in the Additional file 1.
