# Supplementary material for: Finding cancer in mammograms: if you know it’s there, do you know where?
Source: Cogn Res Princ Implic. 2018 Apr 18;3:10. doi: 10.1186/s41235-018-0096-5 (PMC5904219; doi:10.1186/s41235-018-0096-5)
Supplement: Supplementary file 1 — Figure S1. Detection accuracy: percentage correct for individual radiologists on target present trials for (a) low-density and (b) high-density mammograms on the detection task. The three radiologists that had piloted the experiment previously are illustrated in red. Figure S2. Detection accuracy: percentage correct for individual radiologists on target absent trials for (a) low-density and (b) high-density mammograms on the detection task. The three radiologists that had piloted the experiment previously are illustrated in red. Figure S3. Detection accuracy: sensitivity (d′) for individual radiologists for (a) low-density and (b) high-density mammograms. The three radiologists that had piloted the experiment previously are illustrated in red. Figure S4. Detection and localisation results: percentage correct on the localisation task for individual radiologists on trials when detection was correct for (a) low-density and (b) high-density mammograms. The three radiologists that had piloted the experiment previously are illustrated in red. Chance is 4.4% and adjusted to 9.1% when including the ROA (dotted line) with 95% confidence intervals. Figure S5. Detection and localisation results: percentage correct on the localisation task when a region of acceptance (ROA) around the lesion is included for individual radiologists for (a) low-density and (b) high-density mammograms. The three radiologists that had piloted the experiment previously are illustrated in red. Chance is 4.4% and adjusted to 9.1% when including the ROA (dotted line) with 95% confidence intervals. (DOCX 1002 kb) [file 41235_2018_96_MOESM1_ESM.docx]

**Figure S1** Detection accuracy: percentage correct for individual radiologists on target present trials for (a) Low density and (b) High density mammograms on the detection task. The three radiologists that had piloted the experiment previosuly are illustrated in red.

**Figure S2** Detection accuracy: percentage correct for individual radiologists on target absent trials for (a) Low density and (b) High density mammograms on the detection task. The three radiologists that had piloted the experiment previously are illustrated in red.

**Figure S3** Detection accuracy: sensitivity (d′) for individual radiologists (a) Low density and (b) High density mammograms. The three radiologists that had piloted the experiment previously are illustrated in red.

**Figure S4** Detection and localisation results: percentage correct on the localisation task for individual radiologists on trials when detection was correct for (a) Low density and (b) High density mammograms. The three radiologists that had piloted the experiment previously are illustrated in red. Chance is 4.4% and adjusted to 9.1% when including the ROA (dotted line) with 95% confidence intervals.

**Figure S5** Detection and localisation results: percentage correct on the localisation task when a region of acceptance (ROA) around the lesion is included for individual radiologists for (a) Low density and (b) High density mammograms. The three radiologists that had piloted the experiment previously are illustrated in red. Chance is 4.4% and adjusted to 9.1% when including the ROA (dotted line) with 95% confidence intervals.
